# Supplementary material for: Tree diversity in agroforestry systems of native fine-aroma cacao, Amazonas, Peru
Source: PLoS One. 2022 Oct 13;17(10):e0275994. doi: 10.1371/journal.pone.0275994 (PMC9560059; doi:10.1371/journal.pone.0275994)
Supplement: Attachment — Submitted filename: PONE-D-22-03463_reviewer.docx [file pone.0275994.s002.docx]

The manuscript at present form requires major modification in texts and Result presentation in tabular and or graphical form. Here are the comments that I find should be considered to increase the manuscript to a technically sound work:

1. Why there are two ‘native’ in title of the manuscript? Fine aroma is native or the Cacao is or both?
2. L13–15: Give a suitable research gap before pointing the objectives. The use of adverb in the sentence showed you have explained the research gap. However, ‘Cacao cultivation ..... species in the region’ could not speak the gaps in the knowledge base.
3. L17–18: use the value or value range within parenthesis for moderately low.
4. L20: put value/ value range for similarity within parentheses.
5. L20–22: Please rephrase this portion. It is not understandable that whether you are suggesting further diversification or concluding with present diversified systems at the first half of the sentence.
6. L23: While rephrasing, place this sentence separately before concluding statement such that it can leave a remark for conclusion. At the present form it showed an interpretation of result.
7. L24: I suggest instead of dissimilarity indices, you can use species dissimilarity. Why is ‘especies frutales’ mentioned in the keywords? I could not find same and similar word(s) anywhere in the whole manuscript except in the keywords.
8. L28–30: Whether cacao is the primary crop for which forests were partially cleared or it was for some other cultivations? Give a clearer statement in support of it.
9. L36: Give an appropriate reference to the term ‘conventional cacao system.’
10. L38: How all of a sudden it became a productive system? As just now you termed it to be simplified system requiring agrochemical dependency, which again was not properly managed by smallholders due to high input costs.
11. L39–43: This is good but, statement made- “this tendency towards ..... high input costs” and in this section are discrete.
12. L32–34: Is it reasonable to use the term agroforest/ agroforestry for Cacao system? Because, the system undergoes intense modification in respect to economic priority of the cultivars. Whereas, any agroforest should mimic the tree composition with the native forest or secondary forest. I think ‘Conventional Cacao System’ or ‘Conventional Cacao Agro-ecosystem’ would be more appropriate.
13. L56–57: Add more recent reference on expected global market demand and price hike on Cacao.
14. L63: Add specified objectives of the study.
15. L74–75: On what basis you are terming them as young, middle age, etc. Also elaborate why <8 years cacao cultivation was deselected?
16. L76: If it is 15×1.5 ha then result is 22.5 not 22.2.
17. It looks that your qudrats (sampling plots) covered an area of 1.5 ha and not 22.2/22.5 ha. Clearly state what was the total area under laid plot not the area that was covered under cultivation? Because, your sampling time i.e., November and December raised questions in covering so much big area under quadrat laying. The section ‘In the afore mentioned ... 22.2 ha sampled’ and ‘Data collection was ... sub-plot was recorded needed rigorous modification to make it clearer to the audience.
18. L84–85: Farmers are mostly aware of vernacular names not scientific name and hence modify the sentence as an appropriately.
19. L89: Give official identification address of ‘KUELAP-UNTRM’ within the parenthesis.
20. L91–92: If you write this way then give the division factor which was used in conversion of CBH to DBH.
21. L93: ‘***Procesamiento y análisis de datos***’ Change it to English.
22. L94–103: Give specific references to each of the analysis performed. For example for richness, for Shannon, Simpson, Margalef, Chao-1 indices, Jaccard and Sorenson.
23. L112: ‘In general, when visualizing the total data...’ Rephrase this portion.
24. L119: ‘Species richness ranged from 1-9 in the plots.’ Table 2 showed Species richness at different aged stands ranged between 9 and 17.
25. L119–120: Modify to – ‘Species richness was the lowest at middle aged (16–29 years) cacao plots.’ Are you observing only Cacao trees in the entire cultivation system? If not, then why you are mentioning middle aged cacao trees? It should be middle aged of the cultivation system.
26. L123–126: ‘Simpson's (1-D) and Shannon's .... middle-age AFS are the least diverse compared to young and old AFS.’ This should be in discussion. Cite results of the diversity indices here.
27. L128: At L121 you have mentioned that total 16 species were observed in the study area; here you are mentioning 454 individuals were distributed among 17 species distributed; and table 3 representing 17 species.
28. Table 3: Give references below table which was used to delineate the species as exotic, native etc. or add a section at method how you have distinguished this?
29. Table 3: What is potential products? Add a section in the manuscript how it was enumerated and how important it is in phytosociological studies?
30. L137–144: ‘The IVI reveals that .... with a low density of individuals.’ This is not proper result writing. You should give the Dr, Fr, Dor, IVI, etc. values representing the valuable species. This section is neither complete result nor complete discussion.
31. How you have calculated IVI? Add a section in the method. Because it looks absolute value, and you are giving percent symbol. Better to use RIVI, this will give more clear expression of observed species.
32. Figure 2: What are Joven, Madura and Adulta? If these are vernacular then give explanation of the term in figure caption.
33. L153–154: ‘there was a total of 8.23 m*ha2-1, 9.96 ..... and old cocoa AFS respectively.’ Should be written as- ‘Total basal area under young, middle-aged and old cacao AFS was observed to be 8.23 m^2^ ha^-1^, 9.96 m^2^ ha^-1^ and 10.95 m^2^ ha^-1^ respectively.
34. ‘m*ha2-1’: Correct it to ‘m^2^ ha^-1^’ across the whole manuscript. The digit 2 should be at superscript of m expressing as squared meter and -1 should be superscripted at ha denoting per hectare.
35. L169: ‘Of the total .... in the three age rang’ change it to – ‘Seven of the total species studied were observed to be common in all the three age categories.’
36. The term ‘reported’ should be changed to observed, found, etc.
37. Figure 4: Change the language of axis title to English.
38. Shannon diversity index (H0) is not the correct expression. It should be H´.
39. L185–191: ‘The Cacao ecological zone .... looks notably promising.’ This section is not relevant for your research. I suggest change these explanations with relevant texts.
40. At the very beginning of discussion avoid citing and linking with earlier reports. Start discussion with some productive phrases relevant to your findings.
41. L200–201: A well-planned shade trees influences positive impact on cacao yield, improved net income together with various ecosystem services subject to implementation of proper management strategies (References).
42. L213: ‘Côte d'Ivoire’, change it to English, if not possible give description within parenthesis.
43. L222: that additionally provides other benefits (name some other benefits in this parenthesis) together with shade.
44. L224–225: Micro-parcelling (give brief description of micro-parcelling) was also a common phenomena observed in the study area because of the presence relatively small cultivable plots (0.5–2 ha).
45. L230–231: ‘since the density ... higher density of cacao plants.’ This explanation is not required.
46. L235­–236: ‘which in young AFS .... so they have a lower density.’ It is a obvious fact so, this portion is not required.
47. L239–240: Jaccard’s and Sorenson’s are similarity indices. They evaluate similarity not dissimilarity. You can evaluate inverse Jaccard or inverse Sorenson for dissimilarity estimation. In such instances you have to explain how the dissimilarity or inverse similarity was evaluated in the methodology.
