# Supplementary material for: Tree diversity in agroforestry systems of native fine-aroma cacao, Amazonas, Peru
Source: PLoS One. 2022 Oct 13;17(10):e0275994. doi: 10.1371/journal.pone.0275994 (PMC9560059; doi:10.1371/journal.pone.0275994)
Supplement: Attachment — Submitted filename: Response to reviewers.docx [file pone.0275994.s003.docx]

I. **Journal Requirements:**
1. Please ensure that your manuscript meets PLOS ONE's style requirements, including those for file naming.

We ensure that the manuscript meets with PLOS ONE´s requirements.

2. In your Methods section, please provide additional information regarding the permits you obtained for the work. Please ensure you have included the full name of the authority that approved the field site access and, if no permits were required, a brief statement explaining why.

The permissions for research and samples collection in agricultural lands is no required.

3. We note that the grant information you provided in the ‘Funding Information’ and ‘Financial Disclosure’ sections do not match.

When you resubmit, please ensure that you provide the correct grant numbers for the awards you received for your study in the ‘Funding Information’ section.

We recheck it.

4. Thank you for stating the following financial disclosure:

“The authors thank the Fondo Nacional de Desarrollo Científico, Tecnológico y de Innovación Tecnológica (FONDECYT) for funding this research through the Contract N° 026-2016 of the "Círculo de Investigación para la Innovación y el fortalecimiento de la cadena de valor del cacao nativo fino de aroma en la zona nor oriental del Perú-CINCACAO" project, executed by the Instituto de Investigación para el Desarrollo Sustentable de Ceja de Selva (INDES – CES), de la Universidad Nacional Toribio Rodríguez de Mendoza de Amazonas. “

Please state what role the funders took in the study.

The authors confirm that: "The funders had no role in study design, data collection and analysis, decision to publish, or preparation of the manuscript."

5. Thank you for stating the following in the Acknowledgments Section of your manuscript:

“The authors thank the Fondo Nacional de Desarrollo Científico, Tecnológico y de Innovación Tecnológica (FONDECYT) for funding this research through the Contract N° 026-2016 of the "Círculo de Investigación para la Innovación y el fortalecimiento de la cadena de valor del cacao nativo fino de aroma en la zona nor oriental del Perú-CINCACAO" project, executed by the Instituto de Investigación para el Desarrollo Sustentable de Ceja de Selva (INDES – CES), de la Universidad Nacional Toribio Rodríguez de Mendoza de Amazonas.”

We note that you have provided additional information within the Acknowledgements Section that is not currently declared in your Funding Statement. Please note that funding information should not appear in the Acknowledgments section or other areas of your manuscript. We will only publish funding information present in the Funding Statement section of the online submission form.

We delete this section.

6. In your Data Availability statement, you have not specified where the minimal data set underlying the results described in your manuscript can be found. PLOS defines a study's minimal data set as the underlying data used to reach the conclusions drawn in the manuscript and any additional data required to replicate the reported study findings in their entirety. All PLOS journals require that the minimal data set be made fully available. For more information about our data policy, please see http://journals.plos.org/plosone/s/data-availability.

Upon re-submitting your revised manuscript, please upload your study’s minimal underlying data set as either Supporting Information files or to a stable, public repository and include the relevant URLs, DOIs, or accession numbers within your revised cover letter. For a list of acceptable repositories, please see http://journals.plos.org/plosone/s/data-availability#loc-recommended-repositories. Any potentially identifying patient information must be fully anonymized.

7. We note that Figure 1 in your submission contain map images which may be copyrighted. All PLOS content is published under the Creative Commons Attribution License (CC BY 4.0), which means that the manuscript, images, and Supporting Information files will be freely available online, and any third party is permitted to access, download, copy, distribute, and use these materials in any way, even commercially, with proper attribution. For these reasons, we cannot publish previously copyrighted maps or satellite images created using proprietary data, such as Google software (Google Maps, Street View, and Earth). For more information, see our copyright guidelines: <http://journals.plos.org/plosone/s/licenses-and-copyright>.

We provide a information about map obtaining in the figure 1 caption.

II. **Review Comments to the Author**

Below we described the actions that we make for response a reviewer recommendations/comment:

1. Why there are two ‘native’ in title of the manuscript? Fine aroma is native or the Cacao is or both?

There was a writing issue. It was corrected.

2. L13–15: Give a suitable research gap before pointing the objectives. The use of adverb in the sentence showed you have explained the research gap. However, ‘Cacao cultivation ..... species in the region’ could not speak the gaps in the knowledge base.

14-15: A new sentence providing a research gap was included.

3. L17–18: use the value or value range within parenthesis for moderately low.

L18: A diversity index value range was provided.

4. L20: put value/ value range for similarity within parentheses.

L21: Value was put.

5. L20–22: Please rephrase this portion. It is not understandable that whether you are suggesting further diversification or concluding with present diversified systems at the first half of the sentence.

L21-24: We were rephrasing this sentence.

6. L23: While rephrasing, place this sentence separately before concluding statement such that it can leave a remark for conclusion. At the present form it showed an interpretation of result.

Done.

7. L24: I suggest instead of dissimilarity indices, you can use species dissimilarity. Why is ‘especies frutales’ mentioned in the keywords? I could not find same and similar word(s) anywhere in the whole manuscript except in the keywords.

L25: We take the recommendation, we use species dissimilarity instead dissimilarity indices. Also, instead “species frutales” we put the translation “fruit species”

8. L28–30: Whether cacao is the primary crop for which forests were partially cleared or it was for some other cultivations? Give a clearer statement in support of it.

L30: We rephrase it

9. L36: Give an appropriate reference to the term ‘conventional cacao system.’.

L38: A reference was provided.

10. L38: How all of a sudden it became a productive system? As just now you termed it to be simplified system requiring agrochemical dependency, which again was not properly managed by smallholders due to high input costs.

We corrected it.

11. L39–43: This is good but, statement made- “this tendency towards ..... high input costs” and in this section are discrete.

L36-41: We rephrase it to better understanding.

12. L32–34: Is it reasonable to use the term agroforest/ agroforestry for Cacao system? Because, the system undergoes intense modification in respect to economic priority of the cultivars. Whereas, any agroforest should mimic the tree composition with the native forest or secondary forest. I think ‘Conventional Cacao System’ or ‘Conventional Cacao Agro-ecosystem’ would be more appropriate.

L35-39: We change the term.

13. L56–57: Add more recent reference on expected global market demand and price hike on Cacao.

L57-59: We changed the sentence and provided more recent information.

14. L63: Add specified objectives of the study.

L64-69: Specific objectives was added.

15. L74–75: On what basis you are terming them as young, middle age, etc. Also elaborate why <8 years cacao cultivation was deselected?

L:88-91: We add an explanation with reference for this selection criteria.

16. L76: If it is 15×1.5 ha then result is 22.5 not 22.2.

L95: We corrected this in all document.

17. It looks that your qudrats (sampling plots) covered an area of 1.5 ha and not 22.2/22.5 ha. Clearly state what was the total area under laid plot not the area that was covered under cultivation? Because, your sampling time i.e., November and December raised questions in covering so much big area under quadrat laying. The section ‘In the afore mentioned ... 22.2 ha sampled’ and ‘Data collection was ... sub-plot was recorded needed rigorous modification to make it clearer to the audience.

It was rephrased to clearer for the audience.

18. L84–85: Farmers are mostly aware of vernacular names not scientific name and hence modify the sentence as an appropriately.

L97-100: We were modifying the sentence.

19. L89: Give official identification address of ‘KUELAP-UNTRM’ within the parenthesis.

L102: Address was provided.

20. L91–92: If you write this way then give the division factor which was used in conversion of CBH to DBH.

L109: The conversion factor was provided.

21. L93: ‘***Procesamiento y análisis de datos***’ Change it to English.

L110: Done

22. L94–103: Give specific references to each of the analysis performed. For example, for richness, for Shannon, Simpson, Margalef, Chao-1 indices, Jaccard and Sorenson.

L114: A references was provided.

23. L112: ‘In general, when visualizing the total data...’ Rephrase this portion.

L137-138: It was rephrased.

24. L119: ‘Species richness ranged from 1-9 in the plots.’ Table 2 showed Species richness at different aged stands ranged between 9 and 17.
    a. L119–120: Modify to – ‘Species richness was the lowest at middle aged (16–29 years) cacao plots.’ Are you observing only Cacao trees in the entire cultivation system? If not, then why you are mentioning middle aged cacao trees? It should be middle aged of the cultivation system.

L144-149: We were rewriting this for better understanding.

25. L123–126: ‘Simpson's (1-D) and Shannon's .... middle-age AFS are the least diverse compared to young and old AFS.’ This should be in discussion. Cite results of the diversity indices here.

L147-149: This sentence was rewrite.

26. L128: At L121 you have mentioned that total 16 species were observed in the study area; here you are mentioning 454 individuals were distributed among 17 species distributed; and table 3 representing 17 species.

L151: We corrected, effectively it was 17 species.

27. Table 3: Give references below table which was used to delineate the species as exotic, native etc. or add a section at method how you have distinguished this?

L103-105: We added a section at methods (L:91-93)

28. Table 3: What is potential products? Add a section in the manuscript how it was enumerated and how important it is in phytosociological studies?

L103-105: We added a sentence explaining how we enumerate the potential products.

29. L137–144: ‘The IVI reveals that .... with a low density of individuals.’ This is not proper result writing. You should give the Dr, Fr, Dor, IVI, etc. values representing the valuable species. This section is neither complete result nor complete discussion.

L59: we rewrite and added IVI values for the most important species to improve this sentence.

30. How you have calculated IVI? Add a section in the method. Because it looks absolute value, and you are giving percent symbol. Better to use RIVI, this will give more clear expression of observed species.

L117-122: We added a section in “materials and methods” to explain how IVI was calculated.

31. Figure 2: What are Joven, Madura and Adulta? If these are vernacular then give explanation of the term in figure caption.

We corrected the language for figure.

32. L153–154: ‘there was a total of 8.23 m*ha2-1, 9.96 ..... and old cocoa AFS respectively.’ Should be written as- ‘Total basal area under young, middle-aged and old cacao AFS was observed to be 8.23 m^2^ ha^-1^, 9.96 m^2^ ha^-1^ and 10.95 m^2^ ha^-1^ respectively.

L177-179: We take the recommendation.

33. ‘m*ha2-1’: Correct it to ‘m^2^ ha^-1^’ across the whole manuscript. The digit 2 should be at superscript of m expressing as squared meter and -1 should be superscripted at ha denoting per hectare.

Done.

34. L169: ‘Of the total .... in the three age rang’ change it to – ‘Seven of the total species studied were observed to be common in all the three age categories.’

L191: We take the recommendation

35. The term ‘reported’ should be changed to observed, found, etc.

Done

36. Figure 4: Change the language of axis title to English.

Done.

37. Shannon diversity index (H0) is not the correct expression. It should be H´.

The expression was checked in hole document.

38. L185–191: ‘The Cacao ecological zone .... looks notably promising.’ This section is not relevant for your research. I suggest change these explanations with relevant texts.

We had decided delete this paragraph.

39. At the very beginning of discussion avoid citing and linking with earlier reports. Start discussion with some productive phrases relevant to your findings.

We rephrasing and reorder this section to improve it.

40. L200–201: A well-planned shade trees influences positive impact on cacao yield, improved net income together with various ecosystem services subject to implementation of proper management strategies (References).

L231-233: We take the recommendation and made the changes.

41. L213: ‘Côte d'Ivoire’, change it to English, if not possible give description within parenthesis.

L206: Changed

42. L222: that additionally provides other benefits (name some other benefits in this parenthesis) together with shade.

L229-230: We added this other benefits

43. L224–225: Micro-parcelling (give brief description of micro-parcelling) was also a common phenomena observed in the study area because of the presence relatively small cultivable plots (0.5–2 ha).

L241-243: We take this recommendation and rewrite the sentence.

44. L230–231: ‘since the density ... higher density of cacao plants.’ This explanation is not required.

We deleted it.

45. L235­–236: ‘which in young AFS .... so they have a lower density.’ It is a obvious fact so, this portion is not required.

We deleted it.

46. L239–240: Jaccard’s and Sorenson’s are similarity indices. They evaluate similarity not dissimilarity. You can evaluate inverse Jaccard or inverse Sorenson for dissimilarity estimation. In such instances you have to explain how the dissimilarity or inverse similarity was evaluated in the methodology.

L125: We added a reference in the “Materials and Methods section”
