# Supplementary material for: Tree diversity in agroforestry systems of native fine-aroma cacao, Amazonas, Peru
Source: PLoS One. 2022 Oct 13;17(10):e0275994. doi: 10.1371/journal.pone.0275994 (PMC9560059; doi:10.1371/journal.pone.0275994)
Supplement: Attachment — Submitted filename: Response Letter.docx [file pone.0275994.s004.docx]

***Dear editor and reviewers, We thank you all for the constructive comments provided. We believe that addressing every single one of them has improved significantly the manuscript.***

***The authors***

***Responses are in italics***

Journal Requirements:

Please review your reference list to ensure that it is complete and correct. If you have cited papers that have been retracted, please include the rationale for doing so in the manuscript text, or remove these references and replace them with relevant current references. Any changes to the reference list should be mentioned in the rebuttal letter that accompanies your revised manuscript. If you need to cite a retracted article, indicate the article’s retracted status in the References list and also include a citation and full reference for the retraction notice.

***Response: All references were check to ensure that is complete and correct.***

***L81 & L307: Reference number [17] was included.***

***4.	Gockowski J, Sonwa D. Cocoa Intensification Scenarios and Their Predicted Impact on CO2 Emissions, Biodiversity Conservation, and Rural Livelihoods in the Guinea Rain Forest of West Africa. Environ Manage. 2011;48: 307–321. doi:10.1007/s00267-010-9602-3 (This reference has been deleted)***

***5.	Asare R, Afari-Sefa V, Osei-Owusu Y, Pabi O. Cocoa agroforestry for increasing forest connectivity in a fragmented landscape in Ghana. Agrofor Syst. 2014;88: 1143–1156. doi:10.1007/s10457-014-9688-3 (This reference change form number5 to number 42)***

***6.	Alemagi D, Duguma L, Minang PA, Nkeumoe F, Feudjio M, Tchoundjeu Z. Intensification of cocoa agroforestry systems as a REDD+ strategy in Cameroon: hurdles, motivations, and challenges. Int J Agric Sustain. 2015;13: 187–203. doi:10.1080/14735903.2014.940705 (This reference has been deleted)***

Reviewer Comments to the Author

Reviewer #1: The manuscript has now improved and has achieved the level to be accepted for publication until the following minor points are addressed:
1. L21–24: Your presented results at L17–21 could not justify the conclusion made. Interpretation of results in the line of conclusion with similar discussion or modification of conclusion in the line of results whichever is feasible for you is suggested.

***Response: L20-24: We rewrite this part of abstract and improve based in your recommendations.***
2. L25: Fruiting species or fruit bearing species

***Response: L25: We change “fruit tree species”***
3. L38: Instead you write, Cacao is a socio-economically viable system....

***Response: L33: We accept this recommendation and change the sentence.***
4. L32–37: Remove this portion: However, farmers .... high input costs; because this is contradictory and it may lead reader to misinterpret conventional cacao and cacao AFS.

***Response: We consider that this recommendation improves the introduction and proceed to remove this portion.***
5. L92: Tree species were identified at the field itself ...

***Response: L87: We accept the recommendation and change the sentence.***
6. L111–112: Reference number 22 (IVI): Why you are citing so much old reference. Our data treatment and representation has improvised to a greater extent since then. You may check following some references for your IVI:
1) https://doi.org/10.1016/j.tfp.2020.100027 (Check IVI calculation and the supplementary table)
2) https://doi.org/10.1007/s11356-022-20329-4 (Check IVI calculation and the supplementary table)

***Response: We cite Curtis and McIntosh (1951) because it is the oldest reference for IVI method development. This method is still valid and has been used in recent studies like Asigbaase et al (2019) and Vera-Velez et al (2019). In addition, the references you mention use the same principle to make the calculations.***

***1:*** [***https://doi.org/10.1371/journal.pone.0210557***](https://doi.org/10.1371/journal.pone.0210557) ***(Asigbaase et al, 2019)***

***2:*** [***https://doi.org/10.1007/s11056-019-09707-y***](https://doi.org/10.1007/s11056-019-09707-y) ***(Vera-Velez et al 2019)***

7. Table 4: No need to put percent symbol against the values given for Dr, Fr, Dor. Because you have already cited in the column heading that the values are in percent. Check the above reference for representing IVI calculation and value representation.

***Response:*** ***We have taken your recommendation into account and have made changes in table 4. Additionally, we change Dr, Fr and Dor by RA, RF and RD respectively (L107-110).***
8. Figure 2: m2 ha-1; here ‘2’ of m2 shoud be superscripted and ‘-1’ of ha-1 should be superscripted.

***Response: We made the suggested changes in figures and text.***
9. L170: m2 ha-1

**Response: We made the suggested change and ensure in whole text.**
